# Anisotropic in-plane thermal conductivity of black phosphorus nanoribbons at temperatures higher than 100 K

**DOI:** 10.1038/ncomms9573

**Published:** 2015-10-16

**Authors:** Sangwook Lee, Fan Yang, Joonki Suh, Sijie Yang, Yeonbae Lee, Guo Li, Hwan Sung Choe, Aslihan Suslu, Yabin Chen, Changhyun Ko, Joonsuk Park, Kai Liu, Jingbo Li, Kedar Hippalgaonkar, Jeffrey J. Urban, Sefaattin Tongay, Junqiao Wu

**Affiliations:** 1 Department of Materials Science and Engineering, University of California, Berkeley, California 94720, USA; 2 Materials Sciences Division, Lawrence Berkeley National Laboratory, Berkeley, California 94720, USA; 3 School for Engineering of Matter, Transport, and Energy, Arizona State University, Tempe, Arizona 85287, USA; 4 Department of Materials Science and Engineering, Stanford University, Stanford, California 94305, USA; 5 State Key Laboratory of Superlattices and Microstructures, Institute of Semiconductors, Chinese Academy of Sciences, Beijing 100083, China; 6 Institute of Materials Research and Engineering, A*STAR (Agency for Science, Technology and Research), 3 Research Link, Singapore 117602, Singapore

## Abstract

Black phosphorus attracts enormous attention as a promising layered material for electronic, optoelectronic and thermoelectric applications. Here we report large anisotropy in in-plane thermal conductivity of single-crystal black phosphorus nanoribbons along the zigzag and armchair lattice directions at variable temperatures. Thermal conductivity measurements were carried out under the condition of steady-state longitudinal heat flow using suspended-pad micro-devices. We discovered increasing thermal conductivity anisotropy, up to a factor of two, with temperatures above 100 K. A size effect in thermal conductivity was also observed in which thinner nanoribbons show lower thermal conductivity. Analysed with the relaxation time approximation model using phonon dispersions obtained based on density function perturbation theory, the high anisotropy is attributed mainly to direction-dependent phonon dispersion and partially to phonon–phonon scattering. Our results revealing the intrinsic, orientation-dependent thermal conductivity of black phosphorus are useful for designing devices, as well as understanding fundamental physical properties of layered materials.

## Abstract (web summary)

Understanding the flow of heat in materials just one or a few atoms thick is vital for harnessing them in compact electronic devices. Here, the authors present the temperature-dependent thermal conductivity of black phosphorus ribbons and demonstrate an intrinsic orientation dependence.

## Graphical abstract

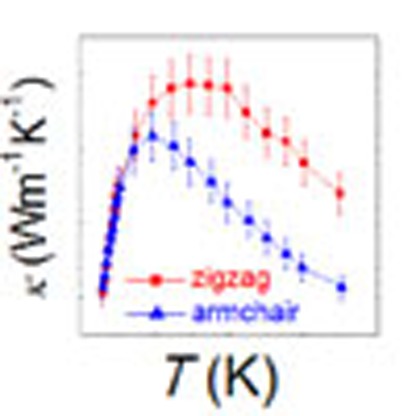

Thermal transport in nanoscale materials attracts increasing research attention because of both intriguing phonon physics at the nanoscale as well as growing importance of heat management in nanoscale devices. Especially, layered nanomaterials provide a new platform for fundamental study, such as two-dimensional electronic effects at the mono- or few-layer limit^1^,^2^, as well as device applications derived from their high flexibility and van der Waals nature of interlayer coupling^3^,^4^,^5^,^6^. It is well known that thermal conductivity in these layered nanomaterials is much lower along the cross-plane direction than the in-plane direction^7^,^8^,^9^,^10^. It is also theoretically predicted that additional anisotropy may exist in their thermal conductivity along different lattice directions in the basal plane^11^,^12^,^13^,^14^,^15^,^16^,^17^. Recent theoretical studies on few-layer graphene nanoribbons expect anisotropic phonon transport along the zigzag (ZZ) and armchair (AC) lattice directions of its honeycomb structure. This anisotropy is not expected in the bulk^11^,^12^,^17^, because it arises mainly from different strengths of boundary scattering at the nanoribbon edges with different chiralities^11^,^17^, that is, specular scattering at the ZZ edge while angle-dependent scattering at the AC edge^17^, an effect that is reduced with increasing nanoribbon width as the material approaches the bulk. However, experimental demonstration of such in-plane anisotropy of thermal conductivity in thin layered materials is lacking, due mostly to technical challenges in sample preparation and measurements.

On the materials side, a new member, black phosphorus (BP), recently joins the family of layered nanomaterials as a promising candidate for electronic, optical and optoelectronic applications. BP has been shown to have some remarkable properties compared with other two-dimensional materials, such as high hole mobility (∼1,000 cm^2^ V^−1^ s^−1^ in field-effect transistors)^6^,^18^,^19^ and tunable direct bandgap from 0.3 eV (bulk) to >1.4 eV (monolayer)^20^,^21^,^22^,^23^. In addition, BP is considered to be a potentially good thermoelectric material^24^,^25^,^26^,^27^. It has been theoretically predicted that BP has opposite anisotropy in thermal and electrical conductivities: electrical conductivity is higher along the AC direction, while thermal conductivity is higher along the ZZ direction^24^,^25^,^28^,^29^,^30^. The anisotropic in-plane thermal transport in BP, in stark contrast to few-layer graphene, is believed to be an intrinsic property^28^,^29^,^30^,^31^, that is, caused by anisotropic phonon dispersion and phonon–phonon scattering rate along the ZZ and the puckered AC directions (Fig. 1a). Therefore, the anisotropy exists not only in the monolayer limit or ribbons but also in multilayer or bulk BP. However, experimental demonstration of this intrinsically anisotropic thermal transport is lacking, owing mostly to the highly preferential growth of single-crystal BP only along the ZZ direction. There exists only one report on the directional thermal conductivity of BP, which was measured at room temperature by Raman thermography^32^.

In this work, we directly measured the in-plane thermal conductivity of single-crystal BP nanoribbons along the ZZ and AC lattice directions. The measurements were carried out in the condition of steady-state longitudinal heat flow, using suspended-pad micro-devices (Fig. 1b), over a wide temperature range from 30 to 350 K. Our results reveal a high anisotropy in thermal conductivity up to a factor of two at temperatures greater than ∼ 100 K. The high anisotropy is attributed mainly to the anisotropic phonon dispersion, and partially to the phonon–phonon scattering. A size effect in the thermal conductivity was also observed from ∼50- to ∼ 300-nm-thick BP nanoribbons in which thinner nanoribbons show lower thermal conductivity. These discoveries not only shed light on phonon physics in this interesting material but also provide important design guidelines in its device applications.

## Results

### Synthesis and characterization of BP crystals

**Figure 1:**
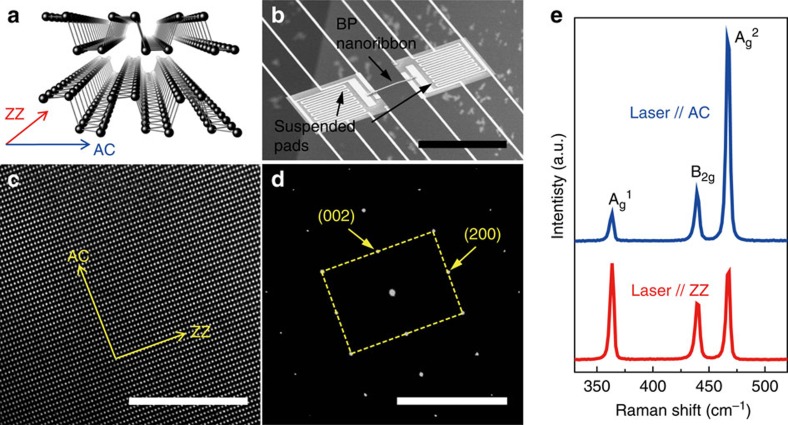
Crystal structure of BP and device structure for the thermal transport experiment. (**a**) Illustration of the crystal structure of BP showing the ZZ and AC axes. ZZ and AC axes correspond to the [100] and [001] direction of the orthorhombic unit cell, respectively (Supplementary Fig. 1a). (**b**) Scanning electron microscopic image of a micro-device consisting of two suspended pads and a bridging BP nanoribbon. Thermal conductivity is measured by transporting heat from the Joule-heated pad to the other pad through the nanoribbon. (**c**) High-resolution transmission electron microscopy lattice image of a BP flake. (**d**) Selected area electron diffraction pattern taken from the area shown in **c**. (**e**) Micro-Raman spectra of a BP flake with laser polarized in parallel to the ZZ and AC axis, respectively. Scale bars, 50 μm (**b**); 10 nm (**c**); 20 nm^−1^ (**d**).

BP bulk crystals were synthesized following a well-developed process, as described in Supplementary Note 1. The synthesized bulk BP (Supplementary Fig. 1a, inset) is comprised of bundles of long crystallites. Powder X-ray diffraction taken from exfoliated BP flakes on a slide glass confirms orthorhombic (Cmca) crystal structure (Supplementary Fig. 1b,c)^33^ without any other phases. Energy dispersive X-ray spectroscopic elemental analysis (Supplementary Fig. 1d) shows only phosphorous element from the crystals. The X-ray diffraction shows exclusively the planes oriented to the [010] direction, proving the layered nature of the crystals. Exfoliated BP flakes were also analysed using a high-resolution transmission electron microscopy with the [010] zone axis (out-of-plane direction), as shown in Fig. 1c. Each flake has identical lattice structure with identical selected area electron diffraction pattern showing the ZZ and AC planes (Fig. 1d), confirming that each flake is a single crystal. The crystallographic orientations agree well with Raman analysis, which utilizes intensity ratio between the Raman peaks of A_g_^1^ and A_g_^2^ phonon modes (Fig. 1e). It is well known that the intensity ratio of the A_g_^2^ mode to the A_g_^1^ mode is the maximum when the laser is polarized in parallel to the AC direction, because the A_g_^1^ and A_g_^2^ modes correspond to atomic vibration along the [010] (out of plane) and [001] (AC, in-plane) directions, respectively^19^,^34^.

### Preparation of BP nanoribbons and suspended-pad micro-devices

**Figure 2:**
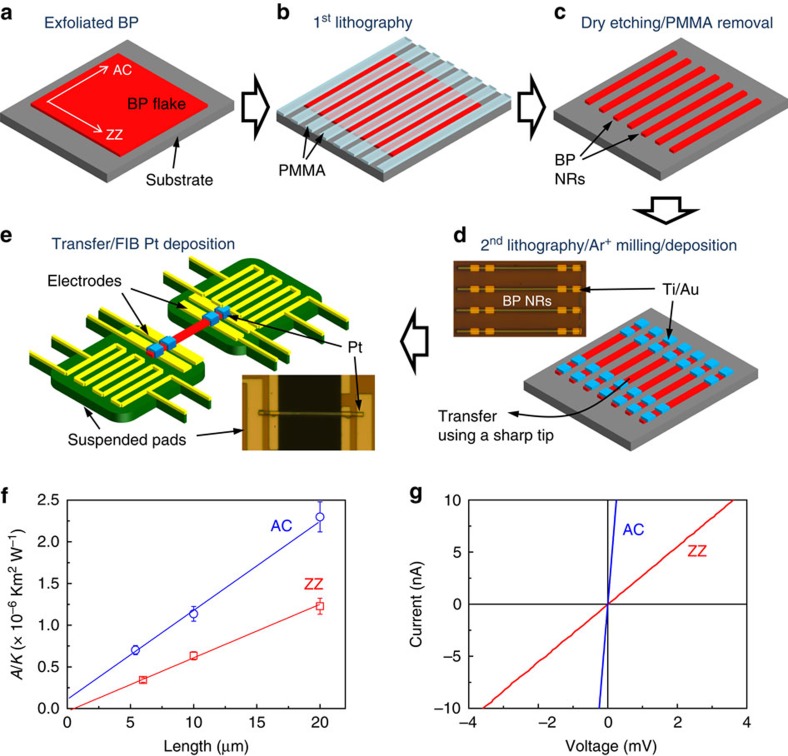
Preparation of BP nanoribbons and nanoribbon-bridged micro-devices. (**a**–**e**) Illustration of the fabrication process. (**a**) Exfoliation of BP flakes onto a SiO_2_ substrate. Crystal directions are identified by Raman analysis. (**b**) Formation of PMMA stripes on the BP flake by the first electron beam lithography (EBL). (**c**) Removal of the exposed BP by dry etching, and removal of the PMMA protecting stripes with acetone, to form BP nanoribbons (NRs). (**d**) Opening up the contact area via the second EBL, Ar^+^ milling of the exposed contact area and electron beam deposition of Ti/Au and lift-off. The Ti/Au layer offers good thermal/electrical contact with BP nanoribbon. Inset shows optical image of BP nanoribbons coated with four Ti/Au contacts resting on the SiO_2_ substrate. (**e**) Dry transfer of a BP nanoribbon onto the micro-device to bridge two suspended pads, and connection of the Ti/Au to the metal electrodes on the pads using FIB Pt bonding. (**f**) Plot of total thermal resistance (1/*K*) at room temperature multiplied by cross-sectional area (*A*) as a function of the nanoribbon length. Error bars include the errors (∼8%) from thermal conductance and sample size measurements. These ribbons have similar thicknesses. The linear relationship extrapolating to nearly zero indicates negligible thermal contact resistance for both ZZ and AC oriented nanoribbons. (**g**) Linear electrical current–voltage curves of the ZZ and AC oriented nanoribbons, measured on the micro-devices. All the measured devices (six devices) show linear *I*–*V* curves.

The thermal conductivity of BP was measured by a steady-state longitudinal heat flow method, using suspended-pad micro-devices as described in Supplementary Note 2 with Supplementary Figs 2–3, as well as in previous reports^35^,^36^. Each pad has two Pt electrodes for four-probe electrical conduction measurements, and a Pt micro-heater/thermometer to control/sense the temperature for thermal conduction measurements. To make BP nanoribbons with proper geometries to bridge the two suspended pads, a top–down micro-fabrication process was used as shown in Fig. 2a–e. First, crystal orientation of BP flakes was identified using Raman analysis; they were then micro-patterned into nanoribbons with desired widths and lengths using electron beam lithography (EBL) and followed by reactive ion etching (see the Method section). Afterwards, a second EBL was applied to define four openings on each BP nanoribbon, followed by *in situ* Ar^+^ milling and Ti/Au deposition with electron beam evaporation. The Ar^+^ milling is essential to remove the surface oxidized layer, which is well known to quickly form when BP is exposed to ambient air. Very mild milling to remove <1 nm of the BP surface was sufficient to obtain a good thermal/electrical contact between the contact metal and the BP nanoribbons. Such contact-ready BP nanoribbons were manually, individually picked up using a sharp probe tip with the aid of a micro-manipulator^37^, and dry transferred onto a suspended-pad micro-device, with careful alignment of the four Ti/Au metal contacts to the four electrodes on the pads. The aligned nanoribbon was subsequently bonded to the electrodes with Pt deposition using a focused ion beam (FIB). Microscopic images for a target BP flake before and after the micro-patterning, and nanoribbons before and after the metal (Ti/Au and Pt) deposition are shown in Supplementary Fig. 4. We note that direct FIB deposition of Pt on the BP, without the pre-formed Ti/Au metal contacts, failed to yield good electrical contacts. To minimize possible damage by the FIB, the Pt deposition was conducted only for connecting the Ti/Au metal contacts to the device electrodes, rather than covering and bonding the entire nanoribbon onto the electrodes. Between each step of the fabrication processes, the BP samples were stored in dry N_2_ atmosphere to maximally prevent surface oxidation. Accumulated time period of exposure to ambient air during the entire fabrication process was estimated to be <15 min, which warrants minimum oxidation of the BP surface, as evidenced by the nano-Auger electron spectroscopic surface analysis (Supplementary Fig. 5). All the thermal and electrical measurements were performed in vacuum (<10^−6^ torr). Figure 2f shows the measured thermal resistance as a function of the length of the BP nanoribbons. The linear fitting and extrapolation yield negligible contact thermal resistance (*y* intercept) between the nanoribbon and the suspended pads for both the ZZ and AC oriented ribbons. Moreover, the Ti/Au/Pt layers make good Ohmic electrical contacts with the nanoribbons, as evidenced by the linear *I*–*V* curves (Fig. 2g).

### Thermal conductivity of BP nanoribbons

We first note that the measured total thermal conductivity (*κ*) in this study is dominated by the lattice (phonon) contribution. Electronic contribution to the thermal conductivity (*κ*_e_) is estimated to be negligible (<0.1% of total) in the measured temperature range. This is calculated from the electrical conductivity (*σ*) measured with four-probe *I*–*V* and converted using the Wiedemann–Franz law, *κ*_e_=*L*_0_σ*T*, where *L*_0_ is the Sommerfeld value of the Lorenz number (Supplementary Fig. 6). We also note that our device geometry and configuration guarantee that the electrical and thermal currents flow along the same crystal direction in the material along the nanoribbon length, which is important to apply the Wiedemann–Franz law.

**Figure 3:**
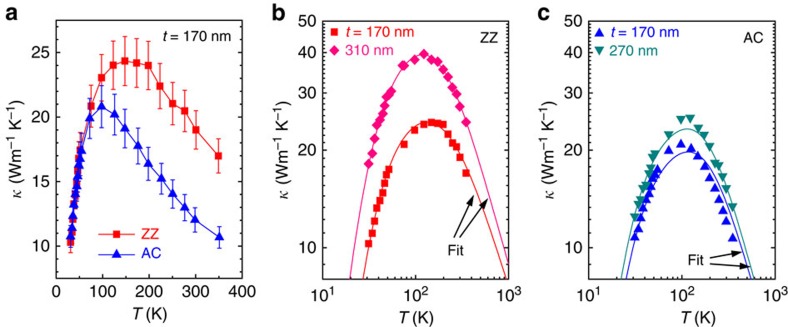
Temperature-dependent thermal conductivity of BP nanoribbons. (**a**) Thermal conductivity (*κ*) versus temperature (*T*) plot of BP nanoribbons axially oriented to the ZZ and AC directions, respectively. Thickness (*t*)/width (*W*) of the ZZ and AC nanoribbons are 170/540 nm and 170/590 nm, respectively. Error bars include the errors (∼8%) from thermal conductance and sample size measurements. (**b**) *κ* versus *T* plots (on logarithmic scale) of ZZ nanoribbons with different dimensions; 170 (*t*)/540 nm (*W*) and 310 (*t*)/540 nm nm (*W*). (**c**) *κ* versus *T* plots (on logarithmic scale) of AC nanoribbons with different dimensions; 170 (*t*)/590 nm (*W*) and 270 (*t*)/420 nm (*W*). The solid lines in **b** and **c** are fitted lines by taking into account various phonon scattering mechanisms (phonon–phonon, impurity and boundary). Lengths of the nanoribbons all exceed 10 μm.

The measured *κ* along the ZZ and AC crystal orientations is compared with each other in Fig. 3a using ZZ and AC nanoribbons with the same thickness and similar widths (<10% difference). It is evident that the ZZ nanoribbon has a higher *κ* than the AC nanoribbon, by as much as ∼7 Wm^−1^ K^−1^ at temperatures above ∼100 K, while both have similar *κ* at lower temperatures between 30 and 100 K. The anisotropy in *κ*, that is, the ratio of *κ* along the ZZ and AC directions, increases with temperature, reaching up to ∼2 at ∼300 K. The increasing anisotropy ratio is attributed to increased contribution from phonon–phonon (Umklapp) scattering, which becomes the dominating scattering mechanism at high temperatures.

The temperature dependency is also observed from other BP nanoribbons that have different dimensions, as shown in Fig. 3b,c. To investigate the anisotropic effect of phonon scattering on the thermal transport in the high-temperature regime, we evaluate scattering times using the relaxation time approximation model with phonon dispersions obtained using the force constants calculated from density function perturbation theory, as detailed in the Methods part and Supplementary Note 3. The branch-dependent group velocities are extracted from the phonon dispersions (Supplementary Fig. 7). The sound velocities are summarized in Supplementary Table 1.

In general, the lattice *κ* along the temperature gradient direction can be expressed as^38^


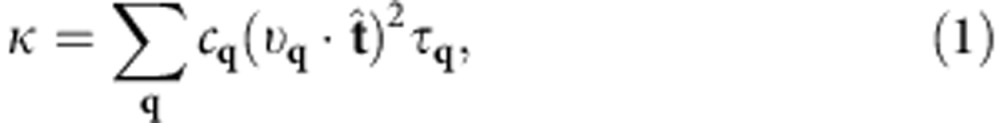


where *c*_**q**_ is the specific heat contributed by the mode with the wavevector **q**, 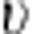 is phonon group velocity, 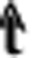 is the unit vector in the direction of temperature gradient, *τ* is the phonon relaxation time and the summation is over the first Brillouin zone of all **q** modes.

Total effects of the phonon–phonon scattering, impurity scattering and boundary scattering are commonly summed up by the Matthiessen's rule, 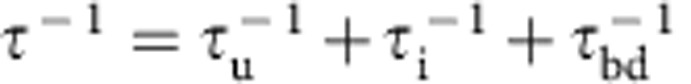. For the phonon–phonon relaxation time (*τ*_u_), the common expression^39^,^40^


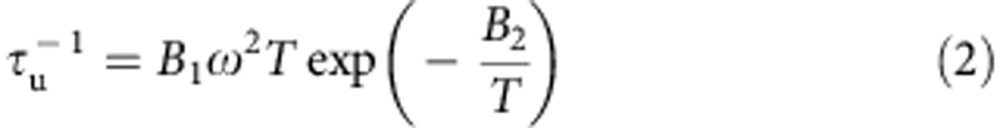


is used, where *B*_1_ and *B*_2_ are fitting parameters and *ω* is phonon frequency. For the impurity scattering, the Klemens' expression 
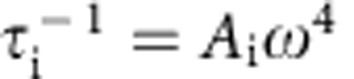
 is used, where *A*_i_ is a parameter that can also be evaluated from the density and species of impurities (generally point defects including native defects such as vacancies and interstitials)^41^. The boundary scattering is determined by a characteristic size and surface roughness of the specimen.

The unknown scattering parameters are usually obtained from fitting *κ* of the bulk, but the bulk *κ* of BP specifically along the ZZ or AC direction has not been reported. Therefore, we first obtain these parameters by fitting *κ* of the nanoribbon with the largest thicknesses in our measurements. This corresponds to 310- and 270-nm thick for the ZZ and AC, respectively. The fitting is validated by the fact that in fitting to the measured *κ* of thinner nanoribbons, good agreements can be obtained by fixing the *A*_i_, *B*_1_ and *B*_2_ at the same values and adjusting only the boundary size 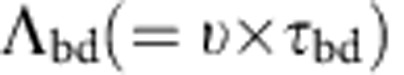 for the thinner ribbons, as shown in Fig. 3b,c. We obtain the following best-fit parameters: *B*_1,ZZ_=5.1 × 10^−19^ sK^−1^, *B*_2,ZZ_=326 K and *A*_i_,_ZZ_=1.0 × 10^−43^ s^3^ for ZZ nanoribbons, and *B*_1,AC_=7.8 × 10^−19^ sK^−1^, *B*_2,AC_=390 K and *A*_i_,_AC_=1.0 × 10^−43^ s^3^ for AC nanoribbons. Among these, the anharmonicity parameter *B*_1_, which is the important parameter at high temperatures, is discussed in the next section. Discussion of the other parameters is presented in Supplementary Note 3.

**Figure 4:**
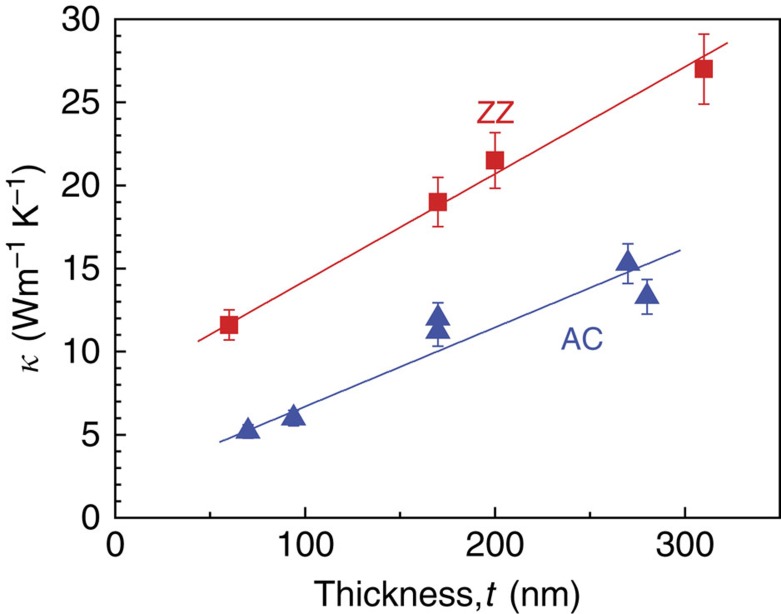
Thickness and orientation-dependent thermal conductivity of BP nanoribbons. Thermal conductivity of ZZ and AC nanoribbons, at 300 K, as a function of thickness. Error bars include the errors (∼8%) from thermal conductance and sample size measurements. The lines show linear fitting of the data to guide the eye.

The minimum flake thickness suitable for our fabrication process is ∼50 nm due to challenges in dry transferring the ultrathin ribbons. BP nanoribbons thinner than 50 nm were not able to be picked up, due to limitation in the sharpness of probe tip. Figure 4 plots room temperature *κ* of the BP nanoribbons as a function of ribbon thickness. *κ* shows a clear thickness dependence along both ZZ and AC directions, decreasing from ∼27 to ∼12 Wm^−1^ K^−1^ in ZZ, and from ∼15 to ∼5 Wm^−1^ K^−1^ in the AC direction, as the thickness varying from ∼300 to ∼50 nm. The anisotropy of *κ* is about 2 over this thickness range. The thickness dependence implies that in this thickness range, surface or boundary scattering is effective even at such a high temperature. The *κ* values measured in this study may differ from predicted *κ* of few-layer BP, due to different heat transport factors, such as evolution of flexural (ZA, out of plane) phonon mode, or more specular boundary scattering with smooth surface in theoretical simulations of few-layer BP. However, the *κ* anisotropy, that is, larger *κ* along the ZZ direction than the AC direction, is in agreement with predictions of monolayer (or few layer) BP. For an example, a recent theoretical work predicts *κ* ∼110 and ∼36 Wm^−1^ K^−1^ at room temperature along the ZZ and the AC direction in monolayer BP^31^.

## Discussion

Compared with the *κ* (∼12 Wm^−1^ K^−1^ at 293 K) of bulk BP reported by Slack in 1965 (ref. 42), our thickest samples have slightly higher values. This is understandable given the fact that their bulk BP was polycrystalline, which can significantly reduce *κ* by grain boundary scattering, cross-layer scattering and averaging of the anisotropy. The crystallite size of the bulk in that work, obtained from fitting to the temperature dependent *κ* below 10 K, is only 10% of the visual crystallite size of the crystal. Therefore, it is reasonable that our thick crystal BP nanoribbons have a higher *κ*. We note that the absolute value of *κ* measured in our study also can be different from a BP single crystal due to possible damage caused during the device fabrication process^43^.

At high temperatures, most acoustic phonon states are excited, which leads to the saturation of specific heat at the Dulong–Petit limit. If the phonon–phonon scattering rate is the same (scattering rate difference will be discussed later), 
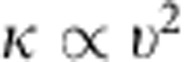
. Thus, *κ* in ZZ direction is higher than AC direction (Fig. 3a) due to the higher group velocities (Supplementary Table 1). If we compare the group velocities of TA1 modes (
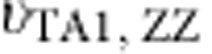
=2,126 m s^−1^ and 
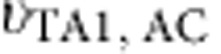
=1,567 m s^−1^, which are close to the results from ultrasonic experiments in literature^44^), we obtain a *κ* anisotropic ratio of 1.85, which is close to our experimental result of ∼2 at high temperatures.

When the temperature drops below 100 K, *κ* becomes close to each other and has a crossover at ∼40 K. The main physical picture for this crossover is freezing out of phonons when temperature drops^45^. At the low-temperature limit, the excited phonon wavevectors (**q**) are small, where the Debye approximation is valid such that 
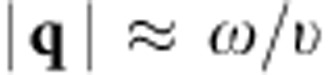
. Comparing the same phonon branch in the ZZ direction to that in the AC direction, the group velocities are all higher in the ZZ direction. Thus, when counting the phonon states in reciprocal **q**-space, the higher group velocity would lead to lower density of states in ZZ direction. Thus, *κ* in the ZZ direction could possibly be lower than *κ* in the AC direction. Thus, in the intermediate temperature, such as between 30 and 75 K, there is a transition regime that the thermal conductivities are close to each other along these two directions.

To investigate which phonons contribute the most to the heat conduction, we evaluated the contributions of two TA branches (out-of-plane TA1, and in-plane TA2) and LA branch in the 170-nm-thick nanoribbons. We find that the TA1 mode contributes the most to the heat conduction among the three acoustic modes. It contributes 53% (48%) of the total *κ* of 19.0 Wm^−1^ K^−1^ (13.5 Wm^−1^ K^−1^) in the ZZ (AC) nanoribbon (Supplementary Table 2). The TA2 mode and LA mode contribute 24% (19%) and 23% (33%), respectively, to the total *κ* in the ZZ (AC) direction for these nanoribbons. In addition, *κ* of TA1 and TA2 branches in the ZZ direction are ∼1.6 and ∼1.8 times of those in the AC direction, respectively, which contributes significant to the anisotropy.

The phonon–phonon scattering, which is the dominant scattering mechanism at high temperatures, are compared by calculating the *τ*_u_ in ZZ and AC directions using the fit parameters. From equation (2), *τ*_u, ZZ_ is ∼1.25 times of *τ*_u, AC_ at room temperature for the identical phonon frequency. Therefore, the different phonon–phonon scattering also contributes in part to the *κ* anisotropy. However, compared with the difference caused by the group velocities (that is, 
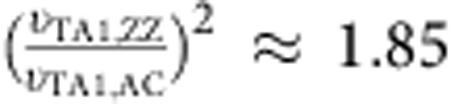
, Supplementary Table 1), the difference in *τ*_u_ is small. Therefore, we attribute the high anisotropy in *κ* mostly to the anisotropic phonon dispersion, and partially to the anisotropic phonon–phonon scattering. In addition, it would also be interesting to know the anharmonic difference, such as the Grüneisen number (*γ*). In general, the anharmonic parameter *B*_1_ is given by 
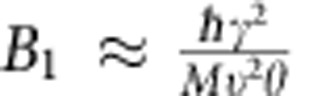
 (ref. 39), where *M* is the average mass of atoms in the crystal, and *θ* is the Debye temperature, which is proportional to group velocity 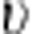. As only *γ* and 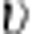 are direction-sensitive parameters, the ratio of *B*_1,ZZ_ and *B*_1,AC_ is 
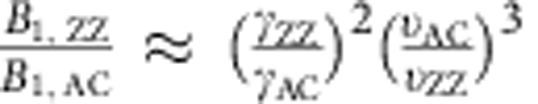
, where 
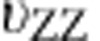
 and 
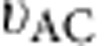
 are the group velocities in ZZ and AC directions. Using the extracted group velocities of TA1 modes (
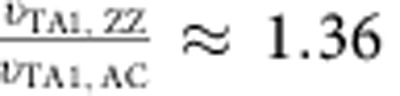
) from the calculated dispersion (the TA1 branch that contributes the most to total *κ*), we obtain 
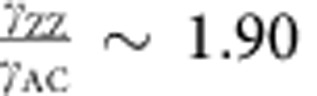
. To the best of our knowledge, this is the first experimental evidence of orientation-dependent anharmonicity in BP.

Due to the relevance of BP for thermoelectrics, we have also measured electrical conductivity (*σ*) and Seebeck coefficient (*S*) along the AC and the ZZ directions, as presented in Supplementary Fig. 8. The room temperature Seebeck coefficients in the AC and ZZ directions are ∼320 and ∼270 μV K^−1^ (*p*-type conductivity), and close to that of the bulk (∼330 μV K^−1^)^26^,^46^. The power factor (=*S*^2^*σ*) of the AC nanoribbon is three times larger than that of the ZZ nanoribbon. *ZT* (=*S*^2^*σT*/*κ*) of the AC nanoribbon (*t*=170 nm) and the ZZ nanoribbon (*t*=200 nm) are ∼0.0036 and ∼0.0006, respectively, at room temperature. This low *ZT* is obviously due to the relatively high *κ* (single-crystalline samples) and low *σ* (undoped samples).

In summary, we discovered a strong in-plane anisotropy in thermal conductivity, up to a factor of two, along the ZZ and AC directions of single-crystal black phosphorous. The BP was prepared in the nanoribbon geometry via a top–down micro-fabrication and the measurements were performed with steady-state longitudinal heat flow using suspended-pad micro-devices. At 300 K, the thermal conductivity decreases as the nanoribbon thickness is reduced from ∼300 to ∼50 nm, but the anisotropy ratio stays around two within the thickness range. Detailed analysis from the relaxation time approximation model shows that the anisotropy in the thermal conductivity originates mostly from phonon dispersion, and partially from phonon–phonon scattering rate, that are both orientation dependent. Our results reveal the intrinsic, orientation-dependent thermal conductivity of BP, which would be important for developing high-performance electronic, optoelectronic and thermoelectric devices using BP, as well as for understanding fundamental physical properties of layered materials approaching the few- or monolayer limit.

## Methods

### Materials preparation and characterization

BP bulk crystals were synthesized from red phosphorus powders with SnI_4_ (American Elements, electronic grade 99.995%) and Sn ingot (Sigma Aldrich) promoters. Please see Supplementary Note 1 for more details. BP flakes were mechanically exfoliated from the synthesized BP bulk crystal, and transferred onto SiO_2_ substrates, using a polydimethylsiloxane stamp (thickness: 1 cm). The morphology and size, crystal structure, composition, phase and crystal orientation of BP flakes were characterized by optical microscopy, SEM, X-ray diffraction, TEM, Energy dispersive X-ray spectroscopic, Auger electron spectroscopic and Raman spectroscopy (with 488 nm laser). For the TEM analysis, selected flakes were transferred from the SiO_2_ substrate to TEM grid using a sharp probe tip by the aid of a micromanipulator.

### Device fabrication for thermal transport experiments

The fabrication of the BP nanoribbons with Ti/Au metal contacts are well described in the main text (Fig. 2a–e). We use the term nanoribbon based on the geometry of the BP samples. For each EBL, PMMA (C4 950) was spin-coated (4,000 r.p.m., 1 min) onto the BP flakes (on SiO_2_ substrate), and baked at 120 °C for 5 min. After the first EBL, dry etching to remove the exposed BP stripes was conducted via reactive ion etching (in mixed gas, 90% SF_6_ and 10% O_2_). The Ti/Au metal was deposited via *in situ* Ar^+^ milling and Ti (10 nm)/Au (70 nm) deposition via electron beam evaporation. Lift-off process was conducted with a gentle shaking in acetone, to avoid fracture of nanoribbons. The final nanoribbons were rinsed thoroughly using isopropyl alcohol.

Suspended-pad micro-devices were used to simultaneously measure the thermal and electrical transport properties of the nanoribbons. Pt lines were patterned on SiN_*x*_ based, suspended pads and flexural arms. Each pad has two arms to measure the electrical conductance of the nanoribbon bridging the two pads, and four arms to measure the electrical conductance of pre-patterned Pt micro-heater/thermometers on the pads for thermal conductivity measurements. A prepared nanoribbon was transferred to bridge the suspended pads using a sharp probe tip. Pt/C composite was deposited using FIB to bond the Ti/Au coat of the nanoribbon onto the Pt electrode lines for securing the thermal and electrical contacts. To further secure the contacts, the devices were annealed at ∼370 K for 1 h in vacuum chamber before measurements. To minimize the surface oxidation, all the pristine and fabricated BP samples were stored in a desiccator in dry nitrogen stream with over pressure.

### Thermal/electrical conductivity measurements

Thermal (*K*) and electrical (*G*) conductance of the nanoribbon were measured simultaneously inside a vacuum chamber (<10^−6^ torr). Global temperature (*T*_g_) was controlled by an external electrical heater and a cryogenic cooler which were connected to the sample holder. *G* was measured using the four-probe method, and *K* was determined following a previous reported method. Briefly, *K* was obtained using the relation, 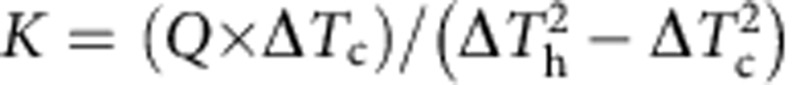, where *Q* is the Joule heating power in the micro-heater, and Δ*T*_h_ and Δ*T*_c_ are the temperature change of the hot and cool pad, respectively. To generate *Q*, a d.c. current was applied to one of the two Pt heater/thermometers. Δ*T* of each pad was evaluated using the resistance change (Δ*R*) of each Pt heater/thermometer on it. The length, width and thickness of the nanoribbon in the study were measured using SEM or AFM, to ultimately determine the thermal conductivity. More details can be found in the Supplementary Note 2. We note that the measured voltage (Keithley 2420A) has <1% of error, hence the mean error for each data point in *I*–*V* curve is negligible compared with the change in voltage, because current was varied in the *I*–*V* measurements. Seebeck coefficient (−Δ*V*/Δ*T*) of the nanoribbon was measured by reading the voltage change in the nanoribbon with varying the temperature difference of the two suspended pads.

### Density functional theory calculation for phonon dispersion

To obtain the phonon dispersion of black phosphorous, we first calculate its atomic structure and the Hessian matrix using the Vienna *ab initio* simulation package^47^,^48^. The projector augmented-wave potentials are used for the ion-electron interactions^49^,^50^, the optB88-vdW exchange-correlation potential is adopted to include long-range dispersion forces^51^,^52^, and the energy cutoff is set to 350 eV. In the density functional theory calculation of the bulk structure, a 10 × 14 × 4 *k*-mesh is used, and the coordinates are fully relaxed until the forces at each atom are <0.001 eV Å^−1^. The calculated lattice constants of the conventional cell are *a*=4.348, *b*=3.329 and *c*=10.495 Å, respectively, consistent with experimental values^53^. In the following density functional perturbation theory calculation^54^, the Hessian matrix is calculated with a 3 × 3 × 3 supercell of the primitive cell and a 5 × 4 × 3 *k*-mesh. On the basis of this Hessian matrix, we extract the force constants and calculate the phonon dispersion using Phonopy^55^.

## Additional information

**How to cite this article:** Lee, S. *et al.* Anisotropic in-plane thermal conductivity of black phosphorus nanoribbons at temperatures higher than 100K. *Nat. Commun.* 6:8573 doi: 10.1038/ncomms9573 (2015).

## Supplementary Material

Supplementary Information Supplementary Figures 1-8, Supplementary Tables 1-2, Supplementary Notes 1-3 and Supplementary References

## References

[b1] Novoselov K. S. *et al.* Two-dimensional gas of massless Dirac fermions in graphene. Nature 438, 197–200 (2005). 16281030 10.1038/nature04233

[b2] Castro Neto A. H., Guinea F., Peres N. M. R., Novoselov K. S. & Geim A. K. The electronic properties of graphene. Rev. Mod. Phys. 81, 109–162 (2009).

[b3] Wang Q. H., Kalantar-Zadeh K., Kis A., Coleman J. N. & Strano M. S. Electronics and optoelectronics of two-dimensional transition metal dichalcogenides. Nat. Nanotechnol. 7, 699–712 (2012). 23132225 10.1038/nnano.2012.193

[b4] Bertolazzi S., Krasnozhon D. & Kis A. Nonvolatile memory cells based on MoS_2_/graphene heterostructures. Acs Nano 7, 3246–3252 (2013). 23510133 10.1021/nn3059136

[b5] Georgiou T. *et al.* Vertical field-effect transistor based on graphene-WS_2_ heterostructures for flexible and transparent electronics. Nat. Nanotechnol. 8, 100–103 (2013). 23263726 10.1038/nnano.2012.224

[b6] Li L. *et al.* Black phosphorus field-effect transistors. Nat. Nanotechnol. 9, 372–377 (2014). 24584274 10.1038/nnano.2014.35

[b7] Slack G. A. Anisotropic thermal conductivity of pyrolytic graphite. Phys. Rev. 127, 694 (1962).

[b8] Minnich A. J. Phonon heat conduction in layered anisotropic crystals. Phys. Rev. B 91, 085206 (2015).

[b9] Balandin A. A. Thermal properties of graphene and nanostructured carbon materials. Nat. Mater. 10, 569–581 (2011). 21778997 10.1038/nmat3064

[b10] Sadeghi M. M., Pettes M. T. & Shi L. Thermal transport in graphene. Solid State Commun. 152, 1321–1330 (2012).

[b11] Xu Y., Chen X., Gu B.-L. & Duan W. Intrinsic anisotropy of thermal conductance in graphene nanoribbons. Appl. Phys. Lett. 95, 233116 (2009).

[b12] Aksamija Z. & Knezevic I. Lattice thermal conductivity of graphene nanoribbons: Anisotropy and edge roughness scattering. Appl. Phys. Lett. 98, 141919 (2011).

[b13] Jiang J.-W., Wang J.-S. & Li B. Thermal conductance of graphene and dimerite. Phys. Rev. B 79, 205418 (2009).

[b14] Liu T.-H., Chen Y.-C., Pao C.-W. & Chang C.-C. Anisotropic thermal conductivity of MoS_2_ nanoribbons: Chirality and edge effects. Appl. Phys. Lett. 104, 201909 (2014).

[b15] Jiang J. W., Zhuang X. & Rabczuk T. Orientation dependent thermal conductance in single-layer MoS_2_. Sci. Rep. 3, 2209 (2013). 23860436 10.1038/srep02209 PMC3713516

[b16] Liu X., Zhang G., Pei Q.-X. & Zhang Y.-W. Phonon thermal conductivity of monolayer MoS_2_ sheet and nanoribbons. Appl. Phys. Lett. 103, 133113 (2013).

[b17] Wei Z., Chen Y. & Dames C. Wave packet simulations of phonon boundary scattering at graphene edges. J. Appl. Phys. 112, 024328 (2012).

[b18] Qiao J., Kong X., Hu Z. X., Yang F. & Ji W. High-mobility transport anisotropy and linear dichroism in few-layer black phosphorus. Nat. Commun. 5, 4475 (2014). 25042376 10.1038/ncomms5475 PMC4109013

[b19] Xia F., Wang H. & Jia Y. Rediscovering black phosphorus as an anisotropic layered material for optoelectronics and electronics. Nat. Commun. 5, 4458 (2014). 25041752 10.1038/ncomms5458

[b20] Zhang S. *et al.* Extraordinary photoluminescence and strong temperature/angle-dependent raman responses in few-layer phosphorene. Acs Nano 8, 9590–9596 (2014). 25188827 10.1021/nn503893j

[b21] Liang L. *et al.* Electronic bandgap and edge reconstruction in phosphorene materials. Nano Lett. 14, 6400–6406 (2014). 25343376 10.1021/nl502892t

[b22] Tran V., Soklaski R., Liang Y. & Yang L. Layer-controlled band gap and anisotropic excitons in few-layer black phosphorus. Phys. Rev. B 89, 235319 (2014).

[b23] Çakır D., Sahin H. & Peeters F. M. Tuning of the electronic and optical properties of single-layer black phosphorus by strain. Phys. Rev. B 90, 205421 (2014).

[b24] Fei R. *et al.* Enhanced thermoelectric efficiency via orthogonal electrical and thermal conductances in phosphorene. Nano Lett. 14, 6393–6399 (2014). 25254626 10.1021/nl502865s

[b25] Zhang J. *et al.* Phosphorene nanoribbon as a promising candidate for thermoelectric applications. Sci. Rep. 4, 6452 (2014). 25245326 10.1038/srep06452 PMC4171703

[b26] Flores E. *et al.* Thermoelectric power of bulk black-phosphorus. Appl. Phys. Lett. 106, 022102 (2015).

[b27] Qin G. *et al.* Hinge-like structure induced unusual properties of black phosphorus and new strategies to improve the thermoelectric performance. Sci. Rep. 4, 6946 (2014). 25374306 10.1038/srep06946 PMC4221793

[b28] Qin G. *et al.* Anisotropic intrinsic lattice thermal conductivity of phosphorene from first principles. Phys. Chem. Chem. Phys. 17, 4854–4858 (2015). 25594447 10.1039/c4cp04858j

[b29] Zhu L., Zhang G. & Li B. Coexistence of size-dependent and size-independent thermal conductivities in phosphorene. Phys. Rev. B 90, 214302 (2014).

[b30] Cai Y. *et al.* Giant phononic anisotropy and unusual anharmonicity of phosphorene: Interlayer coupling and strain engineering. Adv. Funct. Mater. 25, 2230–2236 (2015).

[b31] Jain A. & McGaughey A. J. Strongly anisotropic in-plane thermal transport in single-layer black phosphorene. Sci. Rep. 5, 8501 (2015). 25686917 10.1038/srep08501 PMC4330521

[b32] Luo Z. *et al.* Anisotropic in-plane thermal conductivity observed in few-layer black phosphorus. Preprint at http://arxiv.org/abs/1503.06167v2 (2015). 10.1038/ncomms9572 PMC4634212 26472191

[b33] Kaneta C., Katayamayoshida H. & Morita A. Lattice-dynamics of black phosphorus. I. Valence force-field model. J. Phys. Soc. Jpn 55, 1213–1223 (1986).

[b34] Fei R. & Yang L. Lattice vibrational modes and Raman scattering spectra of strained phosphorene. Appl. Phys. Lett. 105, 083120 (2014).

[b35] Shi L. *et al.* Measuring thermal and thermoelectric properties of one-dimensional nanostructures using a microfabricated device. J. Heat Transfer 125, 881–888 (2003).

[b36] Zhu J. *et al.* Temperature-gated thermal rectifier for active heat flow control. Nano Lett. 14, 4867–4872 (2014). 25010206 10.1021/nl502261m

[b37] Mavrokefalos A., Nguyen N. T., Pettes M. T., Johnson D. C. & Shi L. In-plane thermal conductivity of disordered layered WSe_2_ and (W)_x_(WSe_2_)_y_ superlattice films. Appl. Phys. Lett. 91, 171912 (2007).

[b38] Tye R. P. Thermal Conductivity p17Academic Press, Inc. Ltd. (1969).

[b39] Slack G. A. & Galginaitis S. Thermal conductivity and phonon scattering by magnetic impurities in CdTe. Phys. Rev. 133, A253–A268 (1964).

[b40] AsenPalmer M. *et al.* Thermal conductivity of germanium crystals with different isotopic compositions. Phys. Rev. B 56, 9431–9447 (1997).

[b41] Abeles B. Lattice thermal conductivity of disordered semiconductor alloys at high temperatures. Phys. Rev. 131, 1906 (1963).

[b42] Slack G. A. Thermal conductivity of elements with complex lattices: B, P, S. Phys. Rev. 139, A507–A515 (1965).

[b43] Fu Q., Yang J., Chen Y., Li D. & Xu D. Experimental evidence of very long intrinsic phonon mean free path along the c-axis of graphite. Appl. Phys. Lett. 106, 031905 (2015).

[b44] Kôzuki Y., Hanayama Y., Kimura M., Nishitake T. & Endo S. Measurement of ultrasound velocity in the single crystal of black phosphorus up to 3.3 GPa gas pressure. J. Phys. Soc. Jpn 60, 1612–1618 (1991).

[b45] Toberer E. S., Baranowski L. L. & Dames C. Advances in Thermal Conductivity. Annu. Rev. Mater. Res. 42, 179–209 (2012).

[b46] Warschauer D. M. Black phosphorus as strain gauge and pressure transducer. J. Appl. Phys. 35, 3516 (1964).

[b47] Kresse G. & Furthmuller J. Efficiency of ab-initio total energy calculations for metals and semiconductors using a plane-wave basis set. Comput. Mater. Sci. 6, 15–50 (1996). 10.1103/physrevb.54.11169 9984901

[b48] Appalakondaiah S., Vaitheeswaran G., Lebègue S., Christensen N. E. & Svane A. Effect of van der Waals interactions on the structural and elastic properties of black phosphorus. Phys. Rev. B 86, 035105 (2012).

[b49] Blöchl P. E. Projector augmented-wave method. Phys. Rev. B 50, 17953–17979 (1994). 10.1103/physrevb.50.17953 9976227

[b50] Kresse G. & Joubert D. From ultrasoft pseudopotentials to the projector augmented-wave method. Phys. Rev. B 59, 1758–1775 (1999).

[b51] Dion M., Rydberg H., Schröder E., Langreth D. C. & Lundqvist B. I. Van der Waals density functional for general geometries. Phys. Rev. Lett. 92, 246401 (2004). 15245113 10.1103/PhysRevLett.92.246401

[b52] Klimeš J., Bowler D. R. & Michaelides A. Van der Waals density functionals applied to solids. Phys. Rev. B 83, 195131 (2011).

[b53] Asahina H. & Morita A. Band-structure and optical-properties of black phosphorus. J. Phys. C Solid State Phys. 17, 1839–1852 (1984).

[b54] Baroni S., de Gironcoli S., Dal Corso A. & Giannozzi P. Phonons and related crystal properties from density-functional perturbation theory. Rev. Mod. Phys. 73, 515–562 (2001).

[b55] Togo A., Oba F. & Tanaka I. First-principles calculations of the ferroelastic transition between rutile-type andCaCl2-typeSiO2at high pressures. Phys. Rev. B 78, 134106 (2008).

