# Supplementary material for: Anisotropic in-plane thermal conductivity of black phosphorus nanoribbons at temperatures higher than 100 K
Source: Nat Commun. 2015 Oct 16;6:8573. doi: 10.1038/ncomms9573 (PMC4634207; doi:10.1038/ncomms9573)
Supplement: Supplementary Information — Supplementary Figures 1-8, Supplementary Tables 1-2, Supplementary Notes 1-3 and Supplementary References [file ncomms9573-s1.pdf]

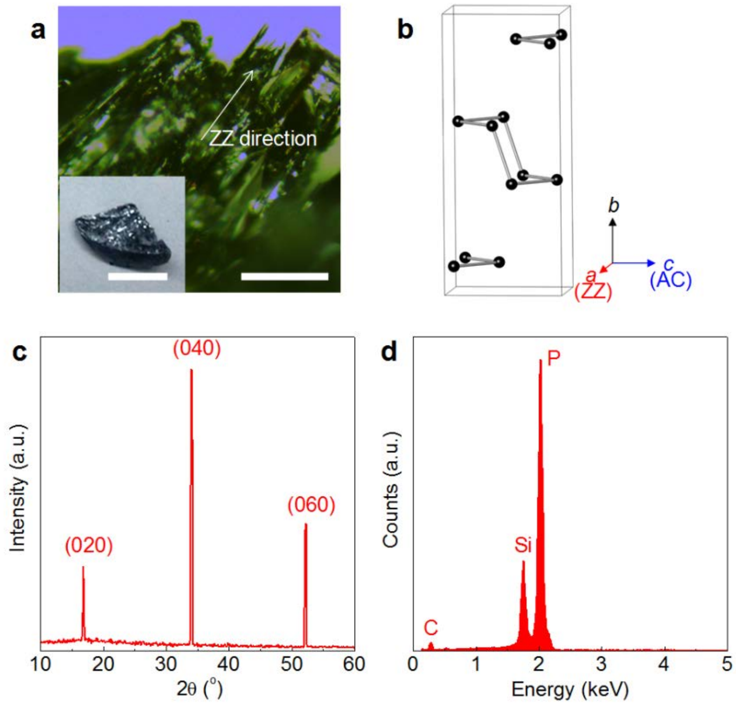

Supplementary Figure 1 │ Characterization of the synthesized BP crystal (a) Optical microscopic image of bulk BP (scale bar: 100 μm). Inset shows as-grown bulk BP specimen (scale bar: 5 mm). (b) Unit cell structure of BP. (c) Powder XRD pattern of BP flakes exfoliated on a glass slide from the bulk specimen. (d) EDS spectrum collected from the BP flakes on a Si substrate. The peaks of Si and C come from the substrate and/or environmental contamination.

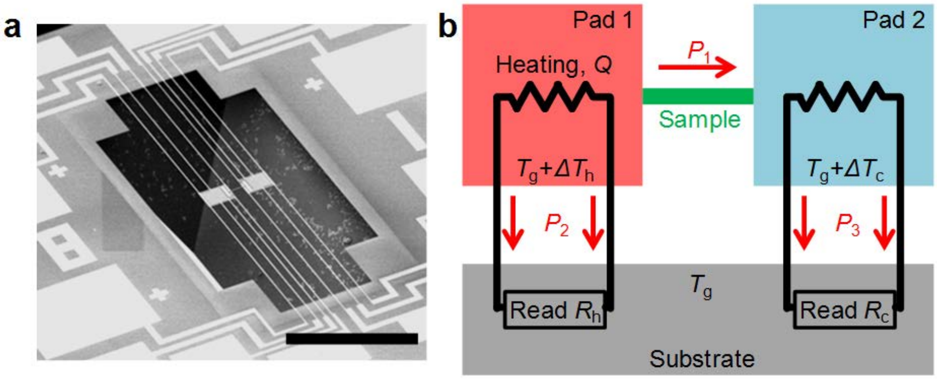

Supplementary Figure 2 │ Principle of the thermal transport measurement using the suspended-pad micro-device (a) SEM image of a suspended-pad device. Scale bar: 200 µm. (b) Schematic illustrating the measurement of thermal conductance (*K*) of a nanoribbon sample bridging the two suspended pads. Not shown are the four electrodes contacting the sample for simultaneous four-probe electrical measurements.

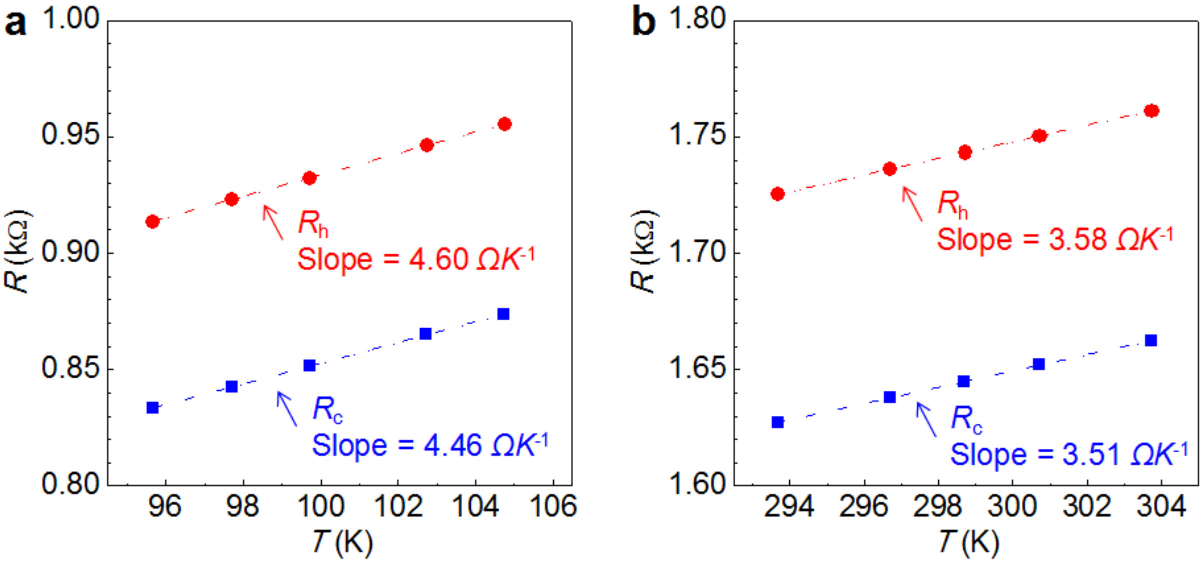

Supplementary Figure 3 │ Resistance (*R*) of micro-heater versus *T* of the hot pad (R_h) and cool pad (R_c) on a micro-device The fitted slopes are used to calibrate the ΔT_h and ΔT_s at the global temperature of (a) ~100 K, and (b) ~299 K. The standard error of the fitted slopes is lower than 0.35%.

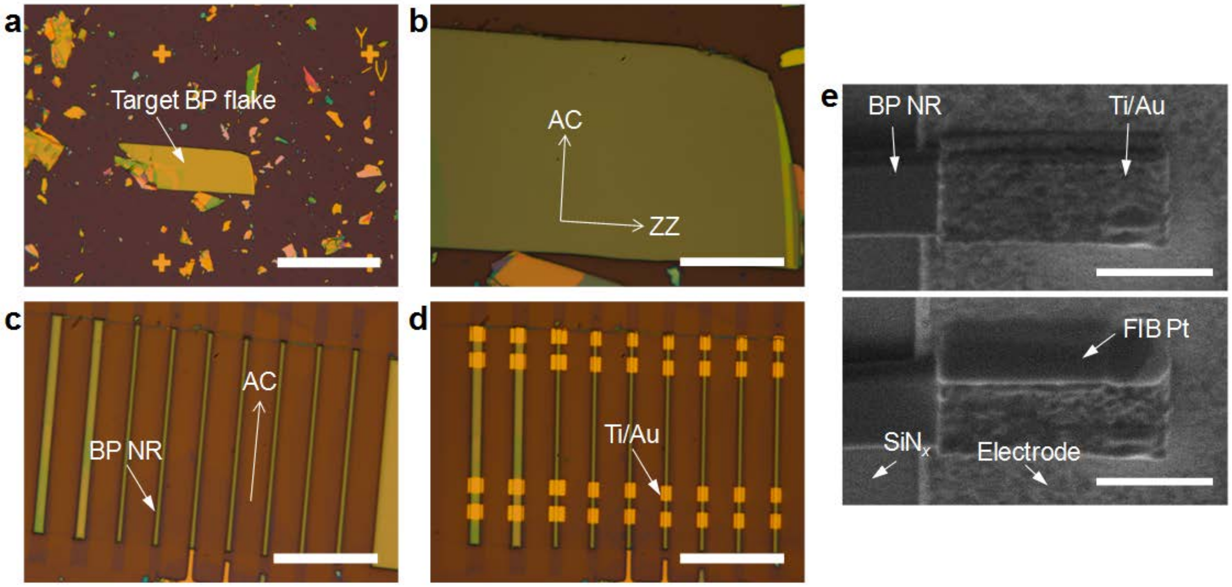

Supplementary Figure 4 | Optical and SEM images taken in the micro-fabrication process (a) As-exfoliated BP flakes on SiO_2 substrate. (b) Magnified image of the target flake indicated in (a). Crystal direction is identified, via Raman analysis, to make AC or ZZ oriented nanoribbons. (c) AC oriented BP nanoribbons made from the target flake via the 1st EBL and RIE processes. (d) The identical nanoribbons after deposition of the Ti/Au metal contacts. (e) SEM images of the contact area between a BP nanoribbon and the electrode on the micro-device, before (upper) and after (lower) FIB Pt deposition. Scale bars: (a) 100 μm, (b-d) 20 μm, and (e) 500 nm.

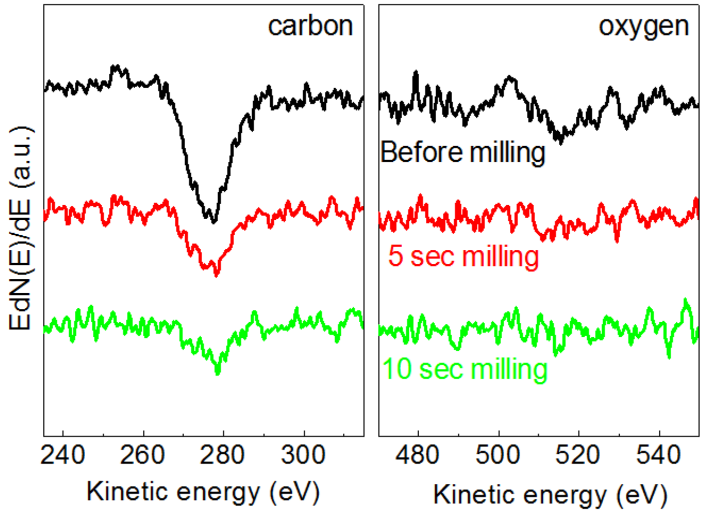

Supplementary Figure 5 | Surface element analysis using nano-AES Auger spectra collected from the surface of a 15 min-air-exposed BP flake. AES equipped with a field emission electron source enables nanoscale chemical analysis at ultra-high vacuum (below 10^{-10} mbar). Barely visible oxygen peak completely disappears after a mild Ar^{+} milling, while the carbon peak which might come from physisorbed organic species still remains, implying that the small amount of oxygen is mainly attributed to physically adsorbed oxygen species at the surface, because removing oxygen from oxidized phosphorus may need more energy. Therefore, it is believed that surface oxidation of the BP sample is negligible. The carbon contamination was also observed to be surface-limited by further sputtering.

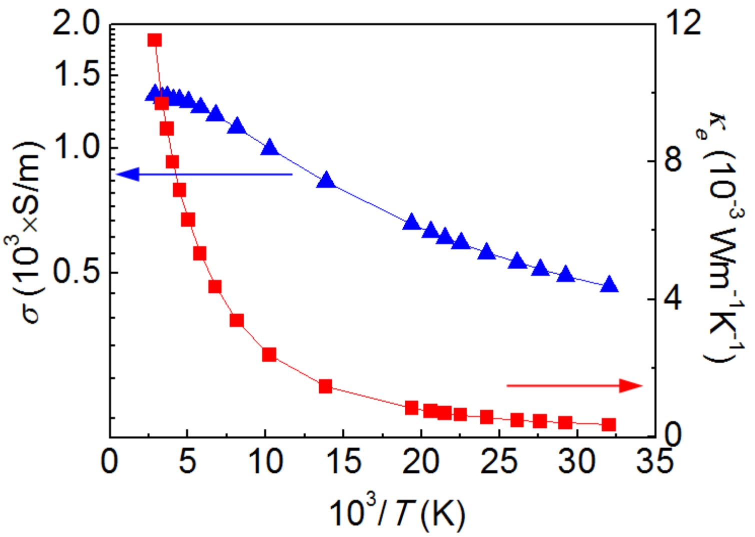

Supplementary Figure 6 | Electrical conductivity of BP nanoribbon Temperature dependent electrical conductivity (σ, left axis, logarithmic scale) of an AC nanoribbon along the AC direction, and corresponding electronic thermal conductivity (right axis), estimated by the Wiedemann-Franz law with the Sommerfeld value of Lorenz number ($L_0 = 2.45 \times 10^{-8}$ WΩK^{-2}). The σ in the AC direction is known to be ~one order of magnitude higher than that in the ZZ direction.[1,2] Electrical conductivity increases as temperature increases. I-V curves are always linear in the investigated temperature range. No size dependence was observed in the electrical conductivity of the BP nanoribbons.

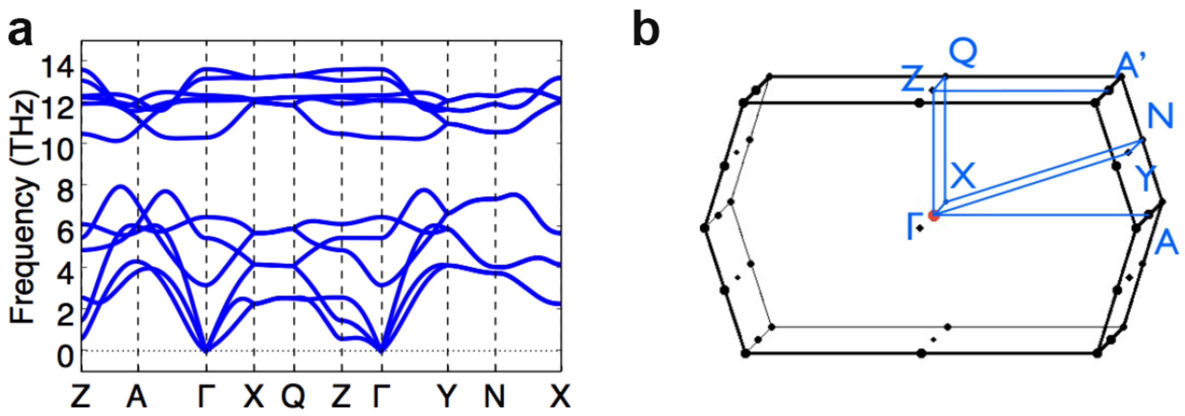

Supplementary Figure 7│Calculated full phonon dispersion (a) Phonon dispersion along high symmetry *q* points. (b) The high symmetry *q* path in the Brillouin zone. The Γ-A and Γ-X directions are corresponding to the ZZ and AC directions, respectively.

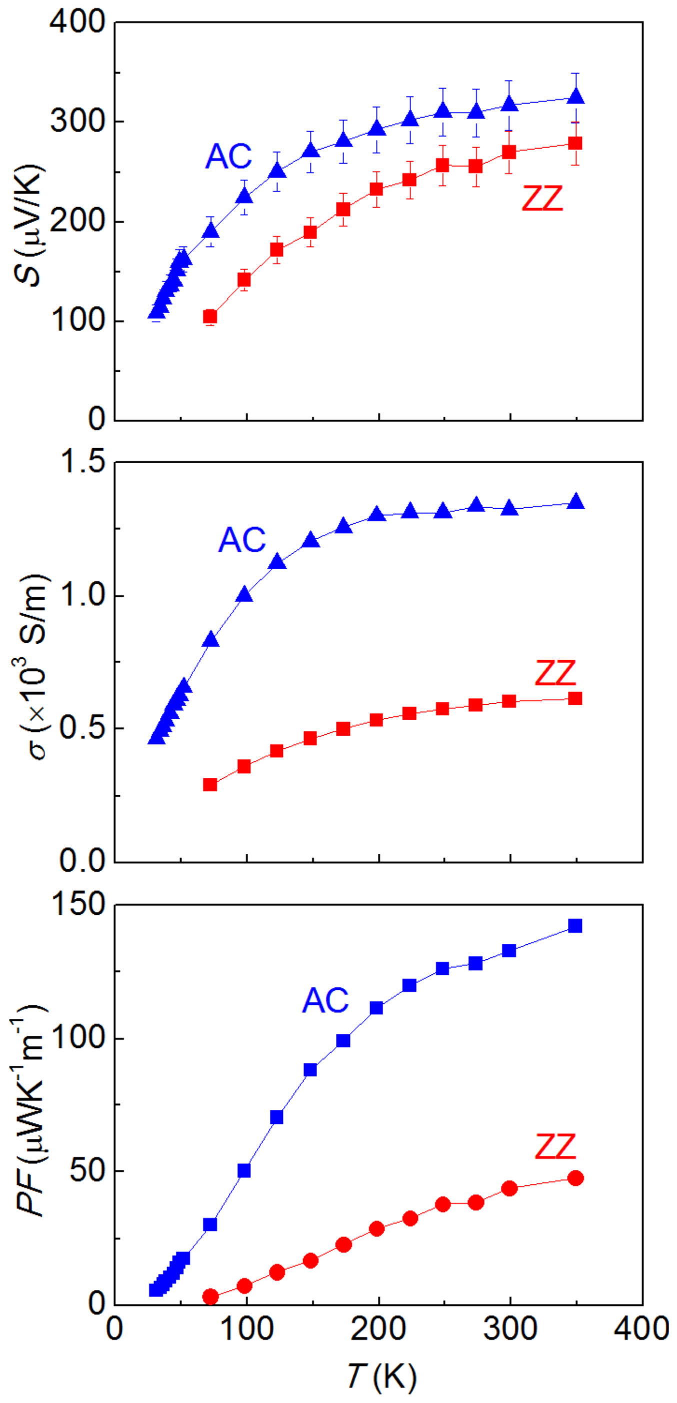

Supplementary Figure 8 | Thermoelectric properties of BP nanoribbons Temperature dependent (a) Seebeck coefficients (S), (b) electrical conductivity (σ), and (c) power factor ($PF = S^2\sigma$) of an AC (t=170 nm), and a ZZ (t=200 nm) nanoribbons. One device for each direction was measured. The ZT ($= S^2\sigma T/\kappa$) of these nanoribbons are ~0.0036 (AC) and ~0.0006 (ZZ) at room temperature. This low ZT is obviously due to the relatively high κ (single-crystalline samples) and low σ (undoped samples).

Supplementary Table 1 │ Sound velocity (υ) in each acoustic phonon mode

Phonon mode	υ_{ZZ} (m sec^{-1})	υ_{AC} (m sec^{-1})	$(\frac{\upsilon_{ZZ}}{\upsilon_{AC}})^2$
Out-of-plane transverse (TA1)	2126	1567	1.85
In-plane transverse (TA2)	4941	4379	1.28
Longitudinal (LA)	7457	5098	2.13
Average	4841	3681	1.75

Supplementary Table 2 │ Thermal conductivity (κ) in each acoustic phonon mode

Phonon mode	κ_{ZZ} (Wm^{-1}K^{-1})	κ_{AC} (Wm^{-1}K^{-1})
Out-of-plane transverse (TA1)	10.1	6.5
In-plane transverse (TA2)	4.6	2.6
Longitudinal (LA)	4.3	4.4
Total	19.0	13.5

Supplementary Note 1 │ Synthesis of bulk BP crystal

BP bulk crystals were synthesized from red phosphorus powders in a sealed tube with SnI_4 (American Elements, electronic grade 99.995%) and Sn ingot (Sigma Aldrich) promoters, innovated by earlier reports by Lange *et.al*.[3] For successful growth of high quality BP bulk crystal, the Sn to SnI_4 ratio is the most critical factor. When the weight ratio was kept to be 1.9~2.1, the largest and cleanest BP crystals was synthesized. In a typical growth process, 20 mg of SnI_4, 40 mg of Sn, and 1 g of red phosphorus was mixed in a silica glass ampoule (15 cm in length and 1.14 cm in diameter) and evacuated to a low pressure (~1×10^{-5} Torr). Synthesis was carried out in a three-zone Lindberg furnace using 1-inch diameter quartz tube. To facilitate the growth, the empty side of the ampoule was set to 50-75 °C below the growth temperature (~700 °C). The furnace was set to 700 °C (ramp time ~3 hr) and kept at this temperature for 3 hr. Then, the ampoule was cooled down to 560 °C in 10 hr, followed by natural cooling down to room temperature. During the natural cooling step, dark orange and red fumes, associated with SnI_4 and red phosphorus, were formed at the colder end. Finally, large shiny BP crystals were formed towards the cold end of the ampoule, well separated from Sn-rich Sn-phosphites, red-phosphorus, and SnI_{4-x} deposits. The BP bulk crystals synthesized following this method is comprised of bundle of axially ZZ-oriented crystallites. The crystal structure, composition, and morphology of the crystallites were carefully investigated as described in the main text. Based on our temperature dependent Raman measurements and purity tests performed by Rutherford Backscattering (RBS), this technique yields higher purity crystals with sharp Raman features

(FWHM <~5cm^{-1}) compared to other commonly used techniques such as white phosphorus to BP conversion at high pressures.[4,5]

Supplementary Note 2 │ Device structure and measurements of thermal conductivity

For the thermal conductance (K_S) and electrical conductance (G) measurements, suspended-pad microdevices (**Supplementary Fig. 2a**) were used. Two suspended SiN_x pads, each supported by six SiN_x arms, are bridged by a nanoribbon, through which heat will flow from the hot pad (Pad 1) to the cool pad (Pad 2). A Pt micro-heater/thermometer (R_{Pt} ~ 2 kΩ) was patterned on each pad to heat up or to sense the temperature of each pad. The arms are also covered with Pt (R_{arm} ~ 1 kΩ each) for electrical reading. The global temperature (T_g) is controlled by an external electrical heater and a cryogenic cooler which are connected to the sample holder. K_S and G of nanoribbons were measured simultaneously inside a vacuum chamber (<10^{-6} Torr). G was measured by using the four-probe method, and K was measured following the steps described below.

When a DC current (I_h) is applied to the micro-heater on Pad 1 (**Supplementary Fig. 2b**), the heat Q generated by the joule heating raises the temperature of Pad 1 by ΔT_h. The heat will transfer to the substrate through the arms (P_2), and to Pad 2 through the sample (P_1), then to the substrate through the arms of Pad 2 (P_3). In steady state, one can write the following equations:

$$Q = P_1 + P_2 \quad (1)$$
$$P_1 = P_3 \quad (2)$$
$$P_1 = K \times (\Delta T_h - \Delta T_c) \quad (3)$$
$$P_2 = n \times K_{arm} \times \Delta T_h \quad (4)$$
$$P_3 = n \times K_{arm} \times \Delta T_c, \quad (5)$$

where K and K_{arm} is the thermal conductance of the sample and the arm, respectively, and n is the number of arms. Therefore,

$$K = (Q \times \Delta T_c)/(\Delta T_h{}^2 - \Delta T_c{}^2) \quad (6)$$

, where Q can be obtained by $Q = I_h{}^2 \times (R_{Pt} + R_{arm})$, and ΔT_h and ΔT_s can be obtained by measuring the resistance of the Pt micro-heater/thermometer on each pad because the temperature coefficient of resistance of Pt is calibrated. An AC current (amplitude < 500 nA) is applied to measure the resistance change of the micro-heater/thermometer. In order to calculate the temperature of each pad, we use the resistance slope ($\Delta R/\Delta T$, where ΔT = 10 K) at each global temperature as shown in **Supplementary Fig. 3**. All the K measured in this study has < 5% error.[6] Finally, the total thermal conductivity κ is obtained considering the geometric factor as $\kappa = K \times l/A$, where A is the cross-sectional area, and l is the length of the nanoribbon.

Thermal conductivity measurement of BP above 350 K is not able using this method, because decomposition of BP begins at around 400 K at the low pressure (< 10^6 Torr) of the chamber.

Supplementary Note 3 │ Anisotropy in density of states of phonons, and fitting parameters

The temperature dependent κ is calculated based on the relaxation-time approximation model, Eq.(1) of the main text. When counting the phonon states for specific heat, we neglected the effect of anisotropy in density of states at the edge of Brillouin zone, because these phonons' contribution (with large wavevectors) to the total κ is relatively small, due to their small υ near

the Brillouin zone boundary. This assumption must be limited to the high temperature regime where most phonon states are excited. We checked it by calculating the phonons' contribution in one third of Brillouin zone ($2/3\ q_{max} < q < q_{max}$) in the ZZ direction. (The phonon frequency range is relatively small in this wavevector regime, even though the wavevector range is large.) These phonons' contribution to the total κ is ~10% at room temperature.

From the fitting to the κ-T data in **Fig. 3b-c** (in main text), we obtained the parameters B_2 (related to the phonon-phonon scattering) and A_i (related to the impurity scattering). The fitting parameters are obtained by fitting the ZZ nanoribbon with thickness 310 nm and AC nanoribbon with thickness 270 nm. After that, we applied the fitting parameters to thinner nanoribbons with the thickness 170 nm (with width 540 nm and 590 nm for ZZ and AC, respectively) to check consistency.

Difference in boundary scattering is less than 5% for the 170 nm-thick ZZ and AC nanoribbons. (The boundary of 220 nm and 210 nm are used for ZZ and AC nanoribbons respectively.) For the impurity fitting parameter A_i, they are close to each other in ZZ and AC directions. Since the impurity scattering are not orientation-dependent, we keep the A_i the same in the AC direction after we obtain it from the ZZ direction. In addition, the anisotropy is strong in the high temperature regime, where the impurity scattering is not the major mechanism of the phonon scattering. For B_2 as the key phonon-phonon scattering parameter, it is empirically close to ~$\theta/3$ at low T. However, Slack showed that $B_2 \approx \theta$ when $T/\theta \approx 1$, and $B_2 > \theta$ when $T/\theta > 1$; *i.e.*, B_2 increases as T/θ increases.[7] In our interested temperature regime, where the phonon-phonon scattering becomes dominant, is near the θ of BP (278.66 K[8] or 400 K[9]), thus our fit parameter $B_2 \approx \theta$ agree well with the trend. We also note that the relaxation time we used here are from empirical expressions. To obtain more accurate phonon scattering information, a full solution to the Boltzmann transport equation is needed.[10]

Supplementary References

1 Keyes, R. W. The Electrical properties of black phosphorus. *Phys. Rev.* **92**, 580-584 (1953).

2 Xia, F., Wang, H. & Jia, Y. Rediscovering black phosphorus as an anisotropic layered material for optoelectronics and electronics. *Nature commun.* **5**, 4458 (2014).

3 Lange, S., Schmidt, P. & Nilges, T. Au_3SnP_7@black phosphorus: An easy access to black phosphorus. *Inorg. Chem.* **46**, 4028–4035 (2007).

4 Bridgman, P. W. Two new modifications of phosphorus. *J. Am. Chem. Soc.* **36**, 1344-1363 (1914).

5 Keyes, R. W. The electrical properties of black phosphorus. *Phys. Rev.* **92**, 580-584 (1953).

6 Zhu, J. *et al.* Temperature-gated thermal rectifier for active heat flow control. *Nano lett.* **14**, 4867-4872 (2014).

7 Slack, G. A. Thermal conductivity of CaF_2, MnF_2, CoF_2, and ZnF_2 crystals. *Phys. Rev.* **122**, 1451-1464 (1961).

8 Qin, G. *et al.* Anisotropic intrinsic lattice thermal conductivity of phosphorene from first principles. *Phys. Chem. Chem. Phys.* **17**, 4854-4858 (2015).

9 Slack, G. A. Thermal conductivity of elements with complex lattices: B, P, S. *Phys. Rev.* **139**, A507-A515 (1965).

10 Broido, D. A., Malorny, M., Birner, G., Mingo, N. & Stewart, D. A. Intrinsic lattice thermal conductivity of semiconductors from first principles. *Appl. Phys. Lett.* **91**, 231922 (2007).
